# Unmasking a Rare Case of Long-Standing Minimal Pericardial Effusion in Dermatomyositis

**DOI:** 10.7759/cureus.59702

**Published:** 2024-05-05

**Authors:** Dibyasundar Mahanta, Debasis Panda, Prakash Kumar, Debasis Acharya, Debasish Das

**Affiliations:** 1 Department of Cardiology, SUM Hospital, Bhubaneswar, IND; 2 Department of Cardiology, All India Institute of Medical Sciences, Bhubaneswar, IND

**Keywords:** minimal, moderate, dermatomyositis, pericardial effusion, unexplained

## Abstract

We report an extremely rare case of long-standing (> six months) minimal pericardial effusion attributed to dermatomyositis. The patient was inadvertently administered antitubercular drug therapy for three months after which the patient developed significant weight loss, extreme anorexia, nausea, and vomiting refractory to conventional management. The key message in the manuscript is that even indolent dermatomyositis can present solely as an unexplained pericardial effusion in an individual.

## Introduction

Asymptomatic pericardial effusion always needs meticulous clinical evaluation to reach a definite diagnosis. The most common cause of pericardial effusion is viral pericarditis, which usually resolves in two weeks. The second most common cause of pericardial effusion in India is tubercular pericardial effusion, which often heals with anti-tubercular therapy. Then comes the autoimmune diseases causing pericardial effusion, the most common being systemic lupus erythematosus (SLE). Index presentation of dermatomyositis with isolated pericardial effusion has not been described in the literature so far. The patient had a clue, i.e., mechanics hand in follow-up, which again pointed toward dermatomyositis. Meticulous clinical examination often reaches a definite diagnosis even if it is an extremely rare entity like dermatomyositis whose incidence is 2.8-3 in 100,000 adults in India [1].

## Case presentation

**Figure 1:**
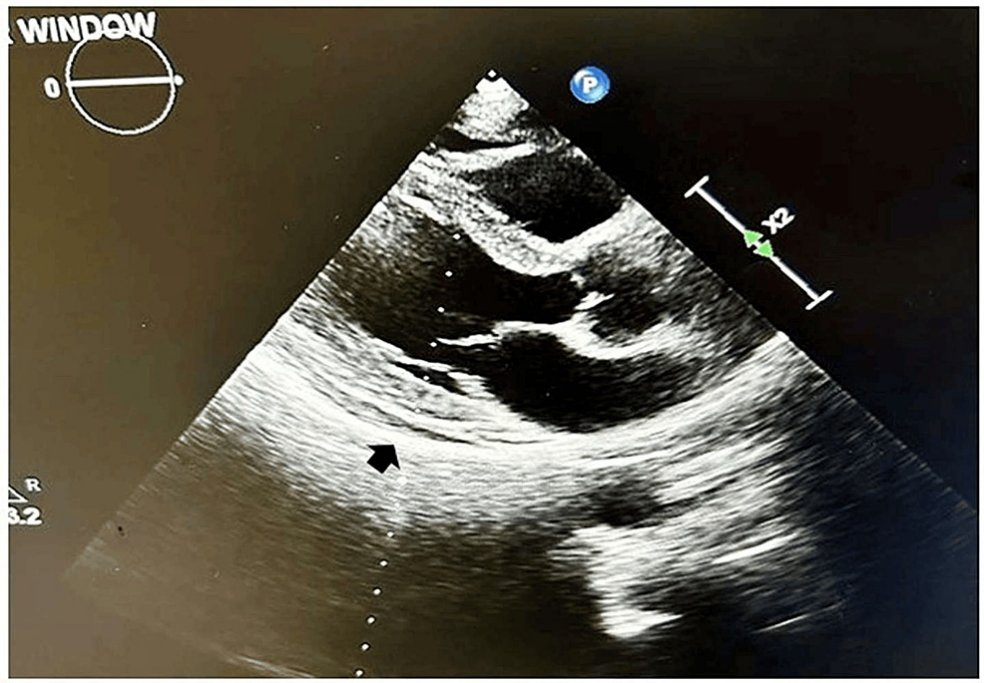
Minimal pericardial effusion

A 60-year-old nondiabetic, nonhypertensive, and nonsmoker presented to the cardiology outpatient department with a history of intermittent precordial pain in deep inspiration for the last six months. He had no history of recent significant weight loss, prolonged dry cough, afternoon fever, or hemoptysis in the past. The patient had been taking the antitubercular drugs for the last three months prescribed by the local physician. The patient was informed by the local physician that he had fluid accumulation around the heart six months back. During the examination, he had a blood pressure of 120/80 mmHg in the right arm supine position and a heart rate of 84 beats per minute. His cardiovascular system examination was within normal limits. His echocardiography revealed the presence of minimal pericardial effusion (Figure 1).

**Figure 2:**
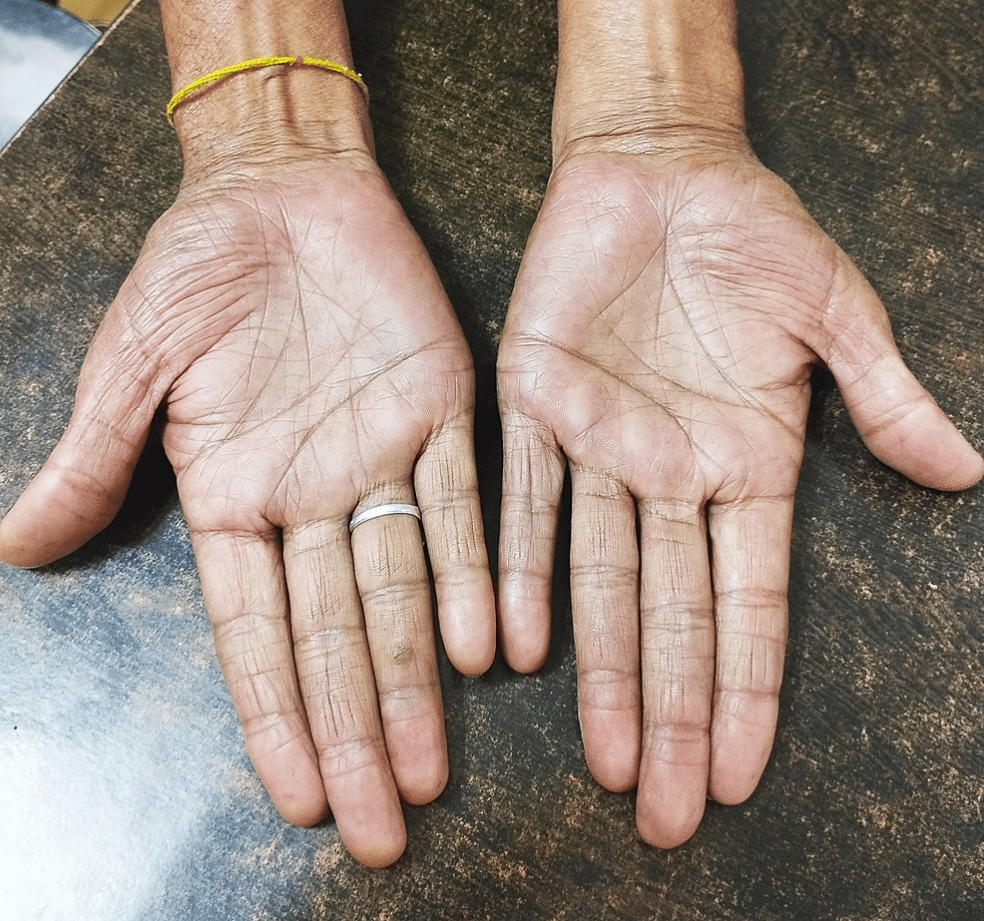
Mechanics hand in dermatomyositis

His total blood count was within normal limits. His serum chemistries were within normal limits except for an elevated erythrocyte sedimentation rate (ESR) of 80 mm/hour. His thyroid profile was within normal limits. As he had minimal pericardial effusion, pericardial fluid could not be sent for analysis. His chest X-ray did not reveal any hilar lymphadenopathy. As he was treated for pericardial tuberculosis, serum interferon-gamma for tuberculosis was sent, which was within normal limits, and his Mantoux test was also negative. His antitubercular therapy was withheld. He was suspected of having an autoimmune disease as the pericardial fluid collection was long-standing and slowly accumulating in nature. His serum antinuclear antibody was elevated, but interestingly, double-stranded DNA was not elevated, ruling out SLE. His serum revealed elevated Jo-1 antibody, suggestive of dermatomyositis. The patient was put on systemic steroid therapy for two months and gradually tapered over one month. Post steroid therapy, there was no pericardial effusion in follow-up echocardiography and he complained of excessive sweating and erythema of both palms mimicking mechanics hand in dermatomyositis (Figure 2).

The patient was advised to attend the rheumatology outpatient department for further immunosuppressive therapy. Our case is an infrequent case where dermatomyositis exclusively presented with minimal pericardial effusion and was successfully treated with steroids. Even a minimal long-standing pericardial effusion can manifest as a multisystem immune disorder in a relatively asymptomatic patient.

## Discussion

We report a rare case of pericardial effusion attributed to dermatomyositis. Initially, there was no clue about the cause of the long-standing minimal pericardial effusion. The patient was inadvertently treated with anti-tubercular therapy because of high ESR and relatively asymptomatic course. Most patients with dermatomyositis have anti-Jo-1 antibody in the serum. The disease usually progresses over time. An interstitial pneumonia-like syndrome occurs in a subset of patients and is associated with high mortality despite aggressive immunosuppressive and corticosteroid therapy [2]. The patient had the mechanics hand in follow-up suggestive of dermatomyositis [3]. The patient had periungual erythema and palmar telangiectasia. He was advised dermatology consultation and was advised topical steroid for the same. The patient had fissuring with hyperkeratotic papules in the fingers and palm suggestive of mechanics hand [4]. In vice versa, most patients with mechanics hand have inflammatory myopathy and most patients have anti-Jo-1 positive antibodies. Besides dermatomyositis, the mechanic's hand is also seen in polymyositis. Anti-Jo-1 antibodies are also known as anti-synthetase antibodies, which are specific for myositis. Dermatomyositis and polymyositis are together known as anti-synthetase syndromes. The patient's chest X-ray did not reveal any feature of interstitial lung disease. The patient dramatically responded to systemic corticosteroid (prednisolone) and he had no pericardial effusion in follow-up after three months. The patient did not have significant muscle symptoms. We started prednisolone 1 mg/kg per day for two months and tapered it over one month. Azathioprine, methotrexate, and cyclophosphamide are the alternatives to steroid therapy [5,6]. Immunoglobulin, plasmapheresis, and whole-body irradiation also play a role in steroid-resistant and refractory cases [7,8]. As the patient did not have significant muscle symptoms like muscle weakness, tenderness, and swelling of muscles, the likelihood of polymyositis in the aforesaid patient was less likely. The presence of skin manifestation pointed more toward dermatomyositis. The patient was able to wake up from a sitting position, excluding significant myositis. Dermatomyositis sometimes presents with a "shawl sign," i.e., violaceous erythema over the neck and back, which was absent in the aforesaid patient. The patient was advised to follow up in the rheumatology and dermatology outpatient department for dermatomyositis. Dermatomyositis presenting exclusively as long-standing pericardial effusion has not been described in the literature so far. Our case is an interesting presentation of cardiac involvement in dermatomyositis, in which the heart is the least commonly affected vital organ in the body as compared to skin, muscles, joints, and lungs.

## Conclusions

We report an extremely rare case of dermatomyositis-induced long-standing minimal pericardial effusion in an otherwise healthy adult. Physicians while trying to unmask the long-standing pericardial effusion should put an eagle eye toward autoimmune diseases, including dermatomyositis, when the patient presents with a pathognomonic mechanics hand.
